# Comparison of in silico strategies to prioritize rare genomic variants impacting RNA splicing for the diagnosis of genomic disorders

**DOI:** 10.1038/s41598-021-99747-2

**Published:** 2021-10-18

**Authors:** Charlie Rowlands, Huw B. Thomas, Jenny Lord, Htoo A. Wai, Gavin Arno, Glenda Beaman, Panagiotis Sergouniotis, Beatriz Gomes-Silva, Christopher Campbell, Nicole Gossan, Claire Hardcastle, Kevin Webb, Christopher O’Callaghan, Robert A. Hirst, Simon Ramsden, Elizabeth Jones, Jill Clayton-Smith, Andrew R. Webster, Genomics England Research Consortium, J. C. Ambrose, P. Arumugam, R. Bevers, M. Bleda, F. Boardman-Pretty, C. R. Boustred, H. Brittain, M. J. Caulfield, G. C. Chan, T. Fowler, A. Giess, A. Hamblin, S. Henderson, T. J. P. Hubbard, R. Jackson, L. J. Jones, D. Kasperaviciute, M. Kayikci, A. Kousathanas, L. Lahnstein, S. E. A. Leigh, I. U. S. Leong, F. J. Lopez, F. Maleady-Crowe, M. McEntagart, F. Minneci, L. Moutsianas, M. Mueller, N. Murugaesu, A. C. Need, P. O’Donovan, C. A. Odhams, C. Patch, D. Perez-Gil, M. B. Pereira, J. Pullinger, T. Rahim, A. Rendon, T. Rogers, K. Savage, K. Sawant, R. H. Scott, A. Siddiq, A. Sieghart, S. C. Smith, A. Sosinsky, A. Stuckey, M. Tanguy, A. L. Taylor Tavares, E. R. A. Thomas, S. R. Thompson, A. Tucci, M. J. Welland, E. Williams, K. Witkowsa, S. M. Wood, Andrew G. L. Douglas, Raymond T. O’Keefe, William G. Newman, Diana Baralle, Graeme C. M. Black, Jamie M. Ellingford

**Affiliations:** 1 North West Genomic Laboratory Hub, Manchester Centre for Genomic Medicine, Manchester University Hospitals NHS Foundation Trust, St Mary’s Hospital, Manchester, UK; 2 Division of Evolution and Genomic Sciences, Neuroscience and Mental Health Domain, School of Biological Sciences, Faculty of Biology, Medicine and Health, University of Manchester, Manchester, UK; 3 Human Development and Health, Faculty of Medicine, University of Southampton, Southampton, UK; 4 Institute of Ophthalmology, UCL, London, UK; 5 Moorfields Eye Hospital NHS Foundation Trust, London, UK; 6 Great Ormond Street Hospital NHS Foundation Trust, London, UK; 7 Manchester Adult Cystic Fibrosis Centre, Manchester University Hospitals NHS Foundation Trust, Manchester, UK; 8 Respiratory, Critical Care and Anaesthesia, UCL Great Ormond Street Institute of Child Health & Great Ormond Street Children’s Hospital & NIHR Great Ormond Street Hospital Biomedical Research Centre, London, UK; 9 Centre for PCD Diagnosis and Research, Department of Infection, Immunity and Inflammation, RKCSB, University of Leicester, Leicester, UK; 10 Wessex Clinical Genetics Service, University Hospital Southampton NHS Foundation Trust, Southampton, UK; 11 Genomics England, London, UK; 12 William Harvey Research Institute, Queen Mary University of London, London, EC1M 6BQ UK

**Keywords:** Disease genetics, Genetics, Clinical genetics, Genetic testing

## Abstract

The development of computational methods to assess pathogenicity of pre-messenger RNA splicing variants is critical for diagnosis of human disease. We assessed the capability of eight algorithms, and a consensus approach, to prioritize 249 variants of uncertain significance (VUSs) that underwent splicing functional analyses. The capability of algorithms to differentiate VUSs away from the immediate splice site as being ‘pathogenic’ or ‘benign’ is likely to have substantial impact on diagnostic testing. We show that SpliceAI is the best single strategy in this regard, but that combined usage of tools using a weighted approach can increase accuracy further. We incorporated prioritization strategies alongside diagnostic testing for rare disorders. We show that 15% of 2783 referred individuals carry rare variants expected to impact splicing that were not initially identified as ‘pathogenic’ or ‘likely pathogenic’; one in five of these cases could lead to new or refined diagnoses.

## Introduction

Pinpointing disease-causing genomic variation informs diagnosis, treatment and management for a wide range of rare disorders, and helps bring an end to the “diagnostic odyssey” undergone by some Mendelian disease patients. Molecular testing, in a healthcare setting, now frequently includes genome and exome sequencing^1–3^. Accurate interpretation and categorization of identified variants remains a key limiting factor despite the availability of guidelines for variant analysis^4,5^.

The capability to interpret variation within the non-coding genome is particularly challenging. Variant interpretation is hindered by the vast number of rare/novel non-coding variants identified in each individual^6,7^, the depleted levels of evolutionary conservation within non-coding regions^8^, and our current lack of understanding of the motifs and interactions that are required for appropriate control of gene expression and regulation^9,10^.

Intragenic genomic variants have the potential to impact splicing^11^, the ubiquitous process in eukaryotic cells of converting nascent pre-messenger RNA (pre-mRNA) molecules into mature messenger RNA (mRNA) which can be transported out of the nucleus to provide a template for protein synthesis. Genomic variation in protein-coding, splice junction and intronic regions of genes can disrupt normal splicing mechanisms and underpin the onset of rare disease^12^. Known mechanisms of splicing disruption include the introduction of cryptic splice sites, disruption of canonical splice acceptor and donor sites, and the disruption of other motifs essential for splicing, e.g. branchpoints and the polypyrimidine tract^12^. The significant impact these events have on transcript and protein structure means such disruption is likely to be pathogenic when in transcripts of genes associated with loss-of-function disease mechanisms. This has already been observed in many disease types, for example in autism and intellectual disability^13^, and rare ophthalmic disorders^14^.

A number of computational tools have been developed to assist in the interpretation of genomic variation impacting splicing, and these tools have been expanded recently to include an array of machine learning tools that have been trained to prioritize splice disrupting variation through diverse means^13,15–18^. Developing standards and recommendations for variants in non-coding regions is an important and emerging area for genome diagnostic services. However, in a similar manner to guidance for missense variants, in silico tools may be used as supporting evidence (*PP3*) to prioritize variants that impact splicing and can thereby assist in variant classification. While the initial reports of these in silico prioritization tools have shown promising results, there is yet to be a formal assessment of their integration, utilization and comparative performance in clinical environments.

The aim of this study was to compare the performance of nine in silico strategies, including eight state-of-the-art algorithms and a consensus approach, to prioritize variants impacting splicing. By applying these findings to known cohorts of variants identified through clinical testing, we aimed to identify the likely diagnostic benefit of routine integration of bioinformatics splicing predictions into diagnostic pipelines.

## Results and discussion

### Functional assessment of variants of uncertain significance identified through clinical genetic testing strategies

**Figure 1:**
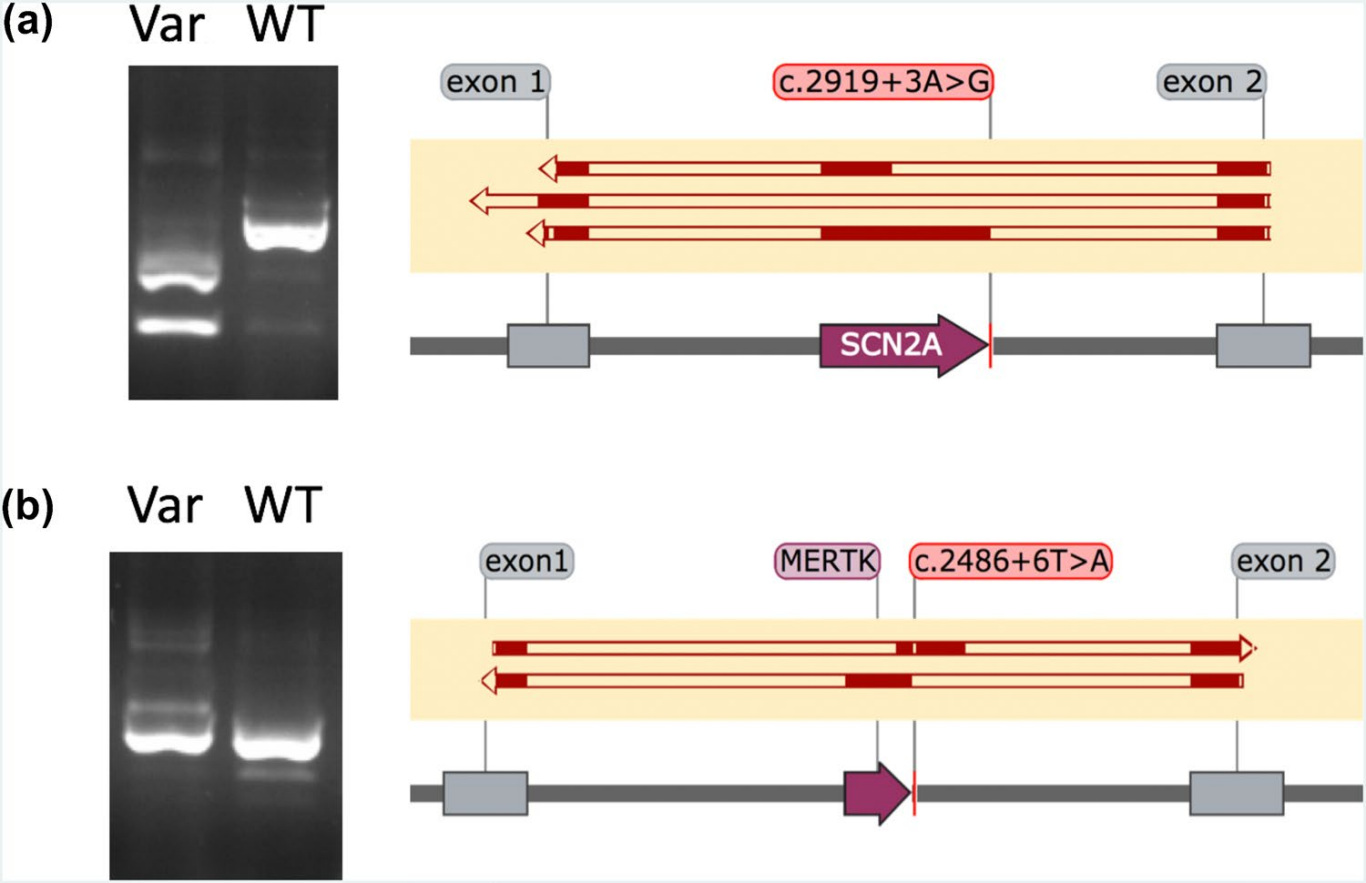
Results from in vitro minigene assays demonstrating multiple consequences as a result of variants proximal to the canonical splice site. Left, gel electrophoresis snapshots of cDNA products amplified from primers designed for control exons within the minigene (*exon 1* & *exon 2*). All prominent bands were cut out and Sanger sequenced. Right, solid red blocks illustrate alignment of sequenced cDNA transcripts to features within the minigene vector: control exons (grey boxes) and inserted exons (purple boxes); (**a**) SCN2A c.2919 + 3A>G, showing complete exon exclusion and exon truncation in minigene vectors containing the c.2919 + 3A>G variant (top two alignments) and normal splicing in minigene vectors containing the WT sequence (bottom alignment). The first resulted in a transcript with a truncated exon, NM_001040142.1:r.2563_2710del, and the second resulted in a complete exon skip, NM_001040142.1:r.2563_2919del. It is noteworthy that if these events were also observed in vivo then they may be considered differently using ACMG criteria; the exon truncation event resulted in a frameshift and introduction of a premature stop codon (*PVS1*), whereas the complete exon skipping event resulted in the inframe removal of 119 amino acids from the transcript (*PM4*); (**b**) MERTK c.2486 + 6T>A, showing a shifting of the exon included in the reading frame in minigene vectors containing the c.2486 + 6T>A variant (top alignment) and normal splicing in minigene vectors containing the WT sequence (bottom alignment). This novel variant is present in two individuals with severe rod-cone dystrophy, and resulted in the simultaneous usage of a cryptic exonic splice acceptor site and a cryptic intronic splice donor site creating a novel exon (chr2:112,779,939–112,780,082, *GRCh37*), and a premature stop codon in the penultimate exon, p.(Trp784Valfs*10). Original images for both *SCN2A* c.2919 + 3A>G and *MERTK* c.2486 + 6T>A are presented in Supplementary Figure S2.

First, we ascertained and performed functional analyses for 249 VUSs to observe their impact on splicing (Supplementary Table S1). To the best of our knowledge, this is the largest set of VUSs that have been functionally interrogated for impact on splicing as part of diagnostic services for individuals with rare disease. All VUSs investigated are in genes where loss-of-function is an expected mechanism of disease causation. Variants had been identified in individuals undergoing genome sequencing and targeted gene panel analysis, with diverse phenotypic presentations including familial susceptibility to breast cancer (MIM #604370), syndromic disorders such as Marfan syndrome (MIM #154700) and isolated inherited retinal disorders such as retinitis pigmentosa (MIM #300029). The approaches for VUS functional analysis are described elsewhere^19^ and in the “Supporting Information”. We observed that 80/249 (32%) of the VUSs significantly impacted splicing, and as a result may be reclassified as ‘likely pathogenic’ according to ACMG guidelines for variant interpretation^4^. This formal reclassification is not conducted as part of this study which focused on the capability of in silico tools to distinguish variants which impact splicing (true positives) and variants which did not impact splicing (true negatives). All VUSs impacted regions outside of canonical splice acceptor and donor sites, and included examples of deeply intronic cryptic splice sites, exonic cryptic splice sites and branchpoint variants. In some cases, functional investigations demonstrated a range of consequences on mRNA splicing (Fig. 1), reinforcing that the precise effect of splicing variants is an important piece of evidence for consideration during clinical variant interpretation that, in the future, may enable refinements in appropriate targeted treatments^20,21^.

### Assessment of in silico prediction strategies to prioritize variants of uncertain significance

**Figure 2:**
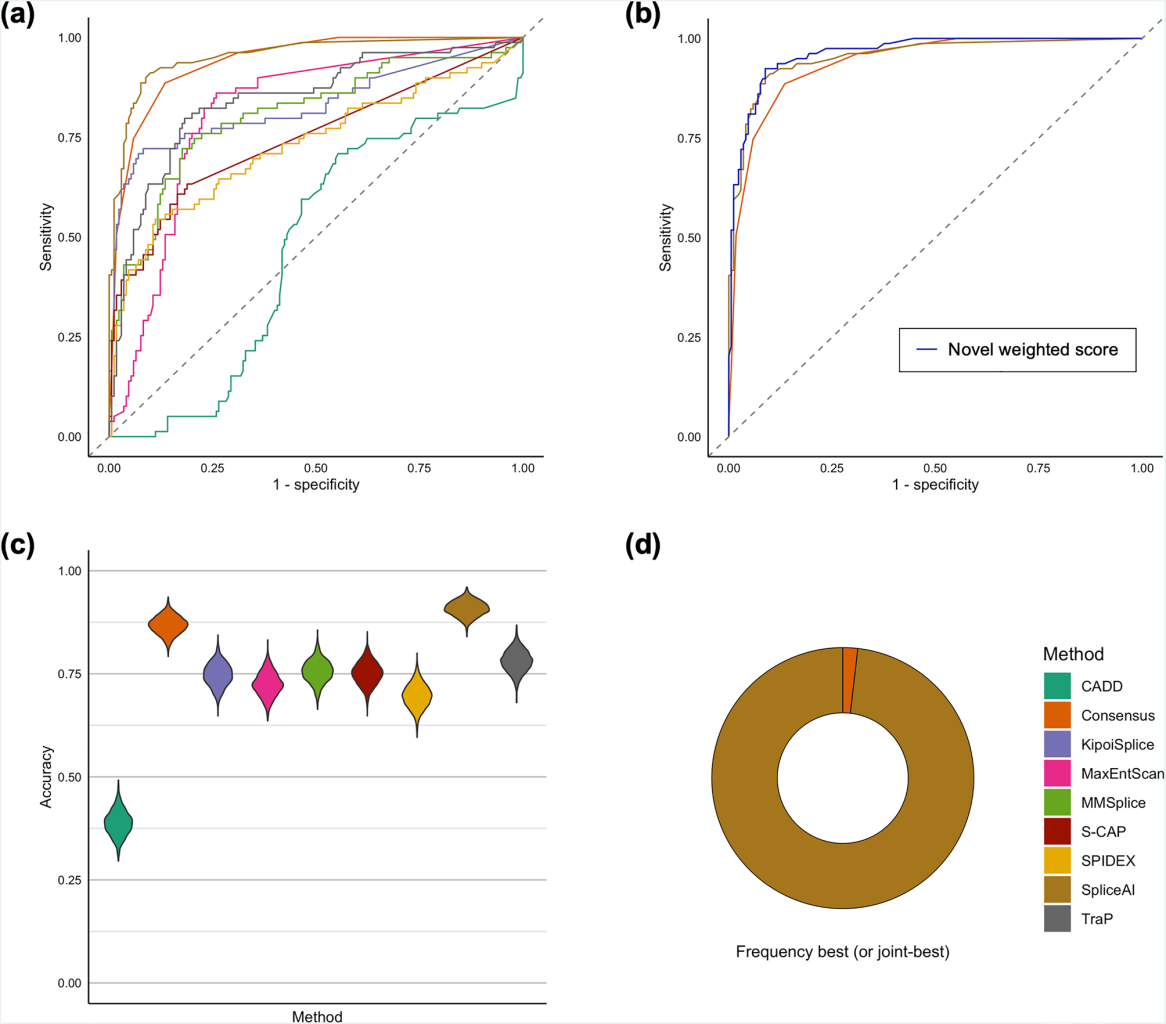
Comparison of in silico strategies to prioritize 249 variants of uncertain significance with functional investigations performed. (**a**) Receiver operating characteristic area under the curve (AUC) comparisons for nine in silico prioritization strategies demonstrating that SpliceAI (AUC = 0.95, 95%CI 0.92–0.97) and a consensus approach (AUC = 0.94, 95% CI 0.91–0.97) outperform other strategies for prioritization; (**b**) AUC comparisons between SpliceAI, a consensus approach and a novel metric, demonstrates that a weighted approach slightly increases accuracy of prioritization over single approaches alone (AUC = 0.96, 95% CI 0.94–0.98); (**c**, **d**) Accuracy comparisons of each in silico prioritization approach across 2000 bootstraps utilizing region-specific pre-defined thresholds: (**c**) Violin plot demonstrating the calculated accuracy of each in silico prioritization approach; (**d**) frequency that each strategy is the best or joint-best performing.

We obtained in silico prediction scores for each of the 249 functionally assessed variants using eight in silico prioritization algorithms (Supplementary Table S1) and calculated sensitivity, specificity and receiver operating characteristic area under the curve (AUC), observing significantly variable performances (Fig. 2). Pairwise statistical comparisons of AUC for the 249 functionally assessed VUSs, after Bonferroni correction for multiple testing, demonstrated that SpliceAI outperformed other single algorithm approaches (Fig. 2; Supplementary Table S2). The AUC analysis for single algorithms calculated the optimal score (based on Youden’s J statistic, as calculated using the pROC software package) for each of the algorithms to distinguish between true positives (80 variants shown to impact splicing in our functional assays) and true negatives (169 variants shown functionally not to impact splicing) in this dataset. We acknowledge that splicing machinery may be influenced by cell-/tissue-specific factors which are outside the scope of assays performed here^22–24^, and variants may have pathogenic impacts on gene expression and/or regulation without any detrimental impact on splicing^8,25–27^. Such factors will influence comparative metrics between algorithms, and future investigations may uncover pathogenic roles for variants reported here. However, the optimal thresholds calculated in light of these limitations for the 249 functionally assessed VUSs in this study are reported in Supplementary Table S3.

Global approaches to variant analysis, as assessed through the AUC, may fail to capture region-specific intricacies in splicing disruption^16^. For example, variants could be sub-divided by their pathogenic mechanism, their effect on pre-mRNA splicing, their predicted molecular consequence or the location of the variant with respect to known splicing motifs, and each of these sub-groups may require different approaches or thresholds for accurate prioritization of pathogenic variation. We therefore predicted variants to be ‘disruptive’ or ‘undisruptive’ according to thresholds pre-defined by the developers of the tools. This included region-specific thresholds for S-CAP and CADD, across six and five different regions, respectively, dependent on variant location in relation to its nearest exon (Supplementary Table S4, see Fig. 3C in Ref.^16^). These regions illustrate if a variant lies in the core splicing dinucleotides, the immediate vicinity of these sites, or at a greater distance. We utilized a single score threshold for tools where region-specific thresholds have not been previously identified (Supplementary Table S4). We compared accuracy of each of the prioritization strategies across 2000 iterations of sampling with replacement. This analysis highlighted differences across the tools and significantly differentiated their ability to accurately predict pathogenicity (Kruskal–Wallis, df = 8, p < 0.0001; Fig. 2c,d). Similar to the AUC analysis, SpliceAI (using a threshold of 0.2) was significantly the best performing strategy across all assessed single algorithms for our set of analyzed VUSs (Kruskal–Wallis, p < 0.0001 for all pairwise comparisons of accuracy between SpliceAI and other tools; Fig. 2c,d).

### Combining in silico tools improves accuracy to identify variants of uncertain significance impacting splicing

To determine if combining one or more of these metrics could achieve greater accuracy than prioritization scores in isolation, we developed a consensus score for each variant which considered the region-specific thresholds for each tool (Supplementary Table S4). The score ranged from 0 to 8 and represented the number of tools for which a variant’s score exceeded the respective threshold. We observed that the consensus approach performed similarly to SpliceAI when assessed through the receiver operating characteristic AUC (Fig. 2; Supplementary Tables S2 and S3). The consensus approach (using a threshold of 4/8 algorithms supporting splicing disruption) also performed more similarly to SpliceAI than other strategies when measuring accuracy across sampling iterations (Fig. 2c), but was less frequently the best performing approach (Fig. 2d). Variability in model accuracy was consistently low across sampling iterations for all tools (Supplementary Table S5). To understand if the relative scores from each algorithm could assist interpretation we developed a novel metric which incorporates weighted scores from the prioritization strategies. This analysis considered the actual score of the variant relative to the maximum score possible from each prediction algorithm (see “Methods”). Of note, the weighted approach considering scores from SpliceAI and a consensus approach performs better than these two approaches in isolation (Fig. 2b; Supplementary Table S3). Although not mutually exclusive and underpowered to detect significant statistical differences in the AUC from this combined analysis—due to marginal gains in accuracy and sample size—this demonstrates the potential utility of combined approaches utilizing combinations of scores to improve accuracy for the identification of variants impacting splicing.

### Integration of in silico strategies to prioritize variants impacting splicing for a large cohort of individuals with rare disease

**Figure 3:**
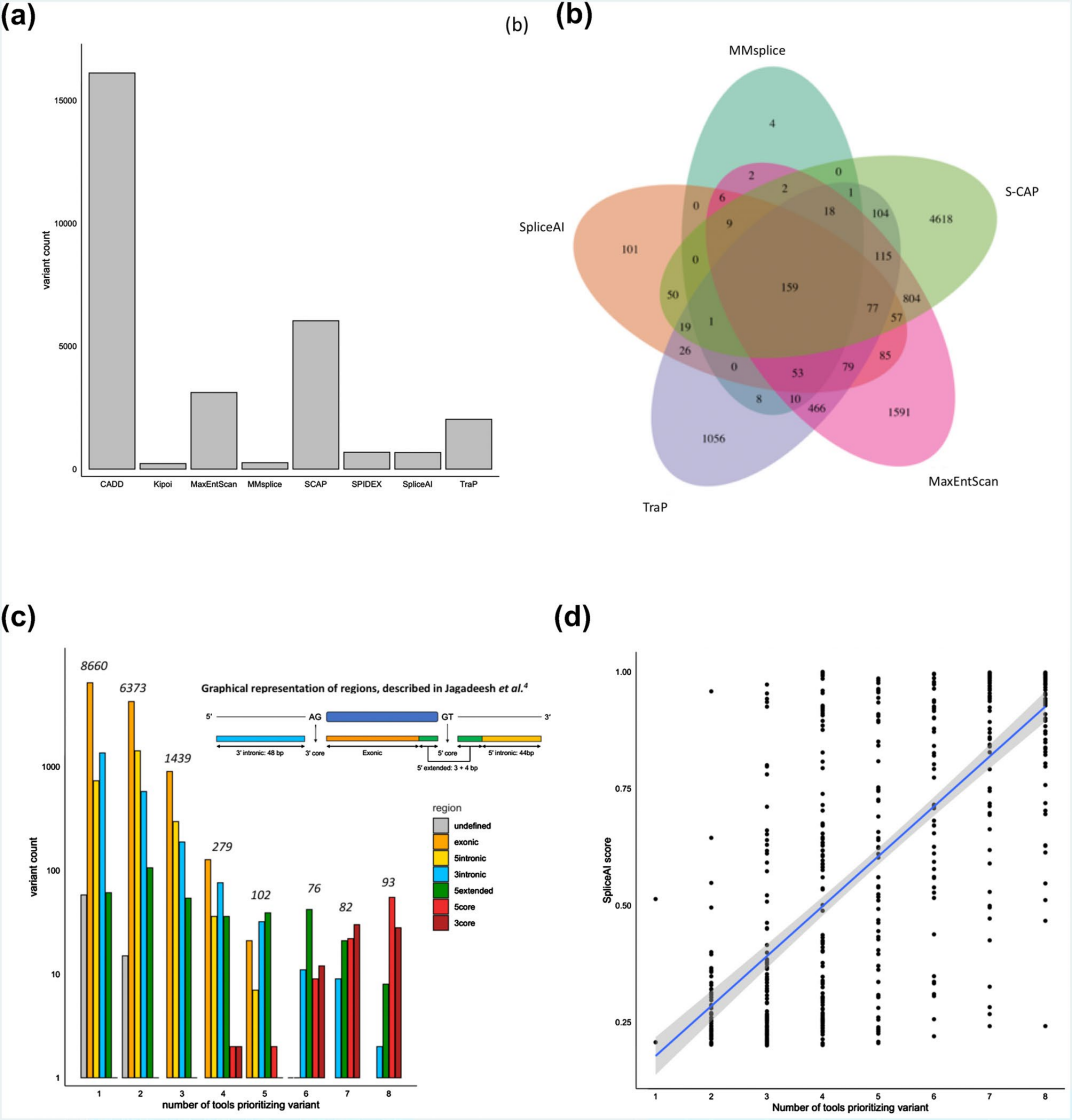
Summary of the overlap and correlations observed between the scores from in silico splicing prediction algorithms for 18,013 unique rare variants identified in a large cohort of 2783 individuals with rare disease undergoing genetic testing, specifically for syndromic and non-syndromic inherited retinal disorders. (**a**) Bar chart showing overall count of unique variants prioritized using pre-defined thresholds for each in silico prediction algorithm; (**b**) Overlap between the unique variants prioritized by the five most correlated in silico prediction tools; (**c**) Grouped bar chart demonstrating the overlap of variants prioritized by each tool segregated by the region of the genome that the variant impacts, as defined by Jagadeesh et al.^16^, demonstrating that variants prioritized by many tools are highly likely to be close the canonical splice sites (5′core, 3′core and 5′extended); (**d**) Correlation between SpliceAI score and the number of additional tools also prioritizing the variant for the 528 unique rare variants prioritized by SpliceAI.

Next, we sought to examine the impact of these approaches on clinical variant analysis. Therefore, we integrated region-specific prioritization strategies (Supplementary Table S4) into an accredited diagnostic service for 2783 individuals with rare diseases^28^. All individuals included in this analysis have received genetic testing for rare disease within the UK National Healthcare Service through a clinically accredited laboratory. We calculated in silico scores for 20,617 variants (of which 18,013 were rare) observed a total of 1,346,744 times in the cohort. We observed substantial variability in the number of rare variants prioritized by each in silico tool (Fig. 3a; Supplementary Table S6) and in the specific variants prioritized by the most correlated in silico splicing tools (Fig. 3b). We observed that while variants which show the highest consensus between in silico splicing tools impact the canonical splice site (Fig. 3c; Supplementary Table S7), 99% (*n* = 17,871) of variants analyzed impact exonic or intronic regions of genes outside of the canonical splice sites. Splicing variants are often considered as a single class of variants and canonical splice site variants are therefore highly susceptible to over-prioritization by in silico tools, as such canonical splice site variants represent the majority (~ 70%) of known pathogenic splicing variants^12,18,29^. Our data further underline the need to develop effective and unbiased strategies for prioritizing variants impacting splicing outside of the canonical splice sites, and this will be especially important for VUSs in known disease genes. Overall, these data demonstrate that different in silico strategies for splicing variant prioritization will alter the burden of variant analysis for clinical scientists. This is an important consideration for the analytical specificity and throughput of diagnostic testing.

To assess the clinical impact of such strategies, we integrated a single prioritization strategy, SpliceAI (using a threshold of 0.2, as above), in parallel to outcomes from routine diagnostic testing. This analysis involved extensive curation of genomic findings for the 2783 referred individuals, all of which were classified in accordance with ACMG guidelines by clinically accredited scientists. We added SpliceAI predictions alongside these analyses and observed that this approach influenced analysis for 420 (15%) individuals receiving genomic testing for rare disease, and prioritized variants that could result in new or refined molecular diagnoses in 81 (3%) cases. Overall, we prioritized 758 variants (528 unique variants) in 646 individuals (23% of cohort) with a range of predicted molecular consequences. Most (99.6%, 526/528) variants were prioritized by at least one other in silico tool (Supplementary Table S8). The strength of the score from SpliceAI correlated highly with prioritization from other in silico tools (Fig. 3d) and differed between regions of genome that were impacted (Supplementary Table S9). We defined prioritized variants as:

- *New*: variant not previously highlighted or reported through diagnostic testing
- *Clarified*: variant previously reported through diagnostic testing but pathogenicity or pathogenic mechanism was unclear
- *Reported*: variant already described or established as ‘pathogenic’ or ‘likely pathogenic’ through diagnostic testing

In this regard, we identified 379 *new* variants in 337 individuals, 87 *clarified* variants in 83 individuals and 292 *reported* variants in 274 individuals. We found most (91%, 697/758) variants to be in genes known as a recessive cause of genetic disease. To understand if these variants impacted normal splicing, we interrogated the GTEx datasets^30^ for individuals carrying these variants in a heterozygous state, identifying 40 carriers of variants prioritized by this analysis. Of these, 21 had suitable RNA-seq datasets available for evaluation, and we were able to clearly observe significant alterations to splicing in four cases (Table 1). Whilst most variants will require bespoke functional investigations to establish precise effects on splicing and protein synthesis, leveraging publicly available datasets for individuals carrying potentially pathogenic rare variants in the GTEx dataset can quickly increase certainty of variant impact and refine clinical variant analysis.

**Table 1:** Metrics obtained from the analysis of GTEx v7 datasets to observe the impact of variants prioritized as splice impacting.

Variant	Gene	Tissue	Metric type	Controls Mean (95% CI)	Cases
20-3899342-G-A	*PANK2*	Fibroblasts	Intron retention	0.12 (0.10–0.14)	0.32
12-88448136-G-A	*CEP290*	Thyroid	NRC	0.15 (0.02–0.27)	0.91
10-73567463-C-T	*CDH23*	Ovary	NRC	0.003 (0–0.01)	0.11
2-110922263-G-A	*NPHP1*	Testis	NRC*	0.51 (0.48–0.55)	0.7

Our analysis identified 4 variants in autosomal recessive genes that were present in a carrier state in individuals in GTEx v7 and had observable impacts on splicing in these individuals. Metrics were calculated from aligned RNAseq datasets from tissues with a transcript per million value > 5 for the gene of interest. *Cases,* individuals within the GTEx dataset carrying prioritized variant. *Controls*, a group of ten randomly selected individuals within the GTEx dataset that do not carry the prioritized variant.

*NRC* normalized read count (described in “Supporting Information”). *switch in usage of two canonical exon junctions in alternative isoforms.

## Conclusions

The incorporation of the prioritization and functional strategies described in this study for variants impacting splicing significantly improved molecular diagnostic services. However, we expect that the true impact of such analysis strategies will be more profound. Targeted next generation sequencing approaches employed within this large cohort ignore deeply intronic regions of genes, which, as shown here (Box 1, Case Example) and in other studies^31–33^, can harbor variants which result in aberrant splicing through the production of novel cryptic exons. The recent availability of genomic datasets within healthcare amplifies the current limitations in interpreting variation within the non-coding genome, particularly in large genome sequencing cohorts. Our findings demonstrate the opportunity to expand bioinformatics analysis to the pre-mRNA regions of known disease genes and provide immediate increases to diagnostic yield. Further, a wide variety of bioinformatics prediction tools continue to be developed, as seen with the recent release of CADD-Splice^34^, and SQUIRLS^35^. As such tools continue to become available, careful analysis of their utility using a framework as described here will allow integration with maximum effect. Future approaches may expand on the consensus model described here through integration of probabilistic models, for example based on Bayesian statistics. Importantly, we demonstrate a requirement to functionally assess variant impact on pre-mRNA splicing as the delineation of the precise effects may be important in considerations for variant pathogenicity. The prioritization and identification of pathogenic variants impacting splicing is therefore an important consideration for diagnostic services and for the development of new targeted treatments.

## Methods

### Patient recruitment and genomic variant dataset generation

All individuals included in this study have provided informed written consent for the analysis of relevant disease-causing genes through tertiary healthcare centers within the UK. All genetic testing procedures have been approved by and are available through the UK National Health Service and were performed in a UK Accreditation Service Clinical Pathology Accredited Medical Laboratory (North West Genomic Laboratory Hub, Manchester, UK; ISO 15189:2012; UKAS Medical reference 9865). All data collected is part of routine clinical care and all investigations were conducted in accordance to the tenets of the Declaration of Helsinki. Analyses to improve genomic services, as reported in this study, have been approved by the North West Research Ethics Committee (11/NW/0421 and 15/YH/0365). Patients reported in individual case reports have provided informed written consent for publication. All individuals with genome sequencing datasets have consented through the Genomics England 100,000 Genomes Project.

Patients were identified with ‘variants of uncertain significance’ (VUSs) according to ACMG guidelines for variant interpretation^4^. Variants were generated through genome sequencing or gene panel sequencing (see “Methods”). All variants investigated are reported in Supplementary Table S1 and their HGVS cDNA nomenclature and genomic co-ordinates (GRCh37 and GRCh38) were validated using VariantValidator^36^.

### Whole genome sequencing

Whole genome sequencing datasets were created through the UK 100,000 Genomes Project^3^, using Illumina X10 sequencing chemistry. Sequencing reads were aligned to build GRCh37 of the human reference genome utilizing Isaac^37^. Small variants were identified through Starline (SNVs and small indels ≤ 50 bp), and structural variants were identified utilizing Manta^38^ and Canvas^39^ (CNV Caller). Variants were annotated and analyzed with the Ensembl Variant Effect Predictor (v75), bcftools and bespoke Perl scripts within the Genomics England secure research embassy.

### Gene panel sequencing

Enrichments were performed on DNA extracted from peripheral blood using Agilent SureSelect Custom Design target-enrichment kits (Agilent, Santa Clara, CA, USA). Enrichment kits were designed to capture known pathogenic intronic variants and the protein-coding regions ± 50 nucleotides of selected NCBI RefSeq transcripts; conditions tested included inherited retinal disease (105 genes or 176 genes), ophthalmic disorders (114 genes), cardiac disorders (72 genes comprised of ten sub-panels) and severe learning difficulties (82 genes). All genes tested and relevant testing strategies are available through the UK Genetic Testing Network (https://ukgtn.nhs.uk/). All samples included in the large cohort analysis were generated through a previously described methodology^40^, and had been completed prior to August 2017. Briefly, samples were pooled and paired-end sequencing was performed using the manufacturer protocols for the Illumina HiSeq 2000/2500 platform (Illumina, Inc., San Diego, CA, USA). Sequencing reads were demultiplexed with CASAVA v.1.8.2. and aligned to the GRCh37 reference genome using Burrows-Wheeler Aligner short read (BWA-short v0.6.2)^41,42^ software before duplicate reads were removed using samtools v0.1.18. The detection and clinical analysis of single nucleotide variants and small insertions/deletions was performed as described previously^40,43^, and in accordance with ACMG guidelines for variant interpretation^4^. During variant analysis, we considered inheritance modes associated with monogenic disorders available in OMIM (https://omim.org/) or PanelApp (https://panelapp.genomicsengland.co.uk/), the zygosity of identified variants, additional variants identified to impact the same gene, phenotype-genotype correlations and scores determined by in silico splicing tools. We identified rare variants within our cohort for prioritization (< 20 heterozygous variants and < 10 homozygous variants) by each of the in silico splicing prediction tools, resulting in 18,013 unique variants and 43,744 total variants (42,281 het and 1463 hom). The region of impact for each rare variant was extracted from S-CAP pre-computed files where available^16^, or determined through Ensembl Variant Predictor (v75) for specified transcripts where unavailable through S-CAP.

### In silico splicing prediction scores

We utilized scores available from CADD^44^, SpliceAI^13^, SPIDEX^18^, S-CAP^16^, MMSplice^17^, TraP^45^, KipoiSplice^46^ and MaxEntScan^47^ to prioritize the 249 variants (we noted on revision that one duplicate variant existed in our dataset). Where multiple scores were available for a variant from the in silico tool, we selected the highest for consideration. To enable comparisons of tool performance and correlation between scores, we converted negative values from SPIDEX, MaxEntScan and MMSplice to positive integers. Whilst these conversions removed directional impact information, i.e. reduced or increased splice site usage, they still reflected the absolute splicing impact of variants. Where scores were unavailable, we assigned the variant a score of 0, i.e. no impact could be predicted. Pre-defined thresholds were applied to determine whether a variant was ‘disruptive’ or ‘undisruptive’ to splicing, as suggested by the authors of the original papers^13,18^, by recent refinements of thresholds^16^, or through nationally recommended guidelines (Supplementary Table S1). A consensus score was generated by considering whether the variant exceeded the threshold of each in silico prediction tool. ROC curves were generated and compared using the pROC package in R. A comparison of accuracy of the tools was performed through 2,000 iterations of sampling with replacement for the 249 samples. Statistical differences in accuracy were identified through the Kruskal–Wallis test in R.

A novel scaled metric was generated for each variant:

$$ score = \sum\limits_{{i = 1}}^{n} {{\raise0.7ex\hbox{${x_{i} }$} \!\mathord{\left/ {\vphantom {{x_{i} } {max_{i} }}}\right.\kern-\nulldelimiterspace} \!\lower0.7ex\hbox{${max_{i} }$}}}  $$

where, *n* = a given combination of the nine prediction strategies, *max* = maximum score from prediction tool *i*, and *x* = variant score from prediction tool *i*. For example, for a variant with a SpliceAI score of 0.85 (the maximum SpliceAI score being 1) and above the threshold of 5/8 tools using the consensus approach:

$$ score = {\raise0.7ex\hbox{${0.85}$} \!\mathord{\left/ {\vphantom {{0.85} 1}}\right.\kern-\nulldelimiterspace} \!\lower0.7ex\hbox{$1$}} + {\raise0.7ex\hbox{$5$} \!\mathord{\left/ {\vphantom {5 8}}\right.\kern-\nulldelimiterspace} \!\lower0.7ex\hbox{$8$}} = 1.475 $$

### RNA investigations

Appropriate functional assays were selected after consideration of gene expression profiles in GTEx (https://gtexportal.org/home/), and the availability of relevant patient samples. We performed assessments on available patient samples or through cell-based minigene assays.

### RNA investigations from patient samples—LCLs and blood

Lymphoblast cell cultures were established for control samples and probands. RNA was extracted using the RNeasy^®^ Mini Kit (Qiagen, UK, Catalogue No. 74104) following the manufacturer's protocol. RNA was extracted from whole-cell blood using the PAXgene™ Blood RNA System Kit (Qiagen, UK. Catalogue No. 762174), following the manufacturer’s protocol for control samples and probands. Extracted RNA was reverse transcribed using the High Capacity RNA to cDNA Kit (Applied Biosystems, UK. Catalogue No. 4387406) following the manufacturer's protocol. Gene specific primers (available on request) amplified relevant regions of the genes being investigated. PCR products were visualized on an agarose gel using a BioRad Universal Hood II and the Agilent 2200 Tapestation. Visualized bands were cut out and prepared for capillary sequencing on an ABI 3730xl DNA Analyzer.

### RNA investigations using cell-based minigene assays

Assays were designed to amplify appropriate genomic regions from patient DNA templates. For variants nearby to wild-type exons, we amplified regions containing one or multiple exons along with flanking ~ 200 intronic nucleotides. For deeply intronic variants we amplified regions containing at least 500 bp of flanking intronic sequence. Primer sequences are available upon request. All regions were amplified from patient DNA templates. For homozygous variants, we also generated a minigene plasmid from a control DNA template. Amplified fragments were checked for size using gel electrophoresis, purified using the QIAquick Gel Extraction kit (Qiagen, UK, Catalogue No. 28706) and then cloned into a customized minigene plasmid (a derivative of the pSpliceExpress vector)^48^ containing an RSV-promoter and two control exons (rat insulin exons 2 and 3) using the NEBuilder^®^ HiFi DNA assembly (NEB, E2621). Amplified fragments were inserted between the two control exons. Plasmids were transformed into competent bacteria (XL-1 blue) and incubated overnight at 37 °C on LB plates containing Carbenicillin. Individual colonies were cultured overnight before isolation of plasmid DNA using the GenElute™ miniprep kit (Sigma-Aldrich, Catalogue No. PLN350). Purified plasmids were Sanger sequenced to confirm successful cloning and identify plasmids containing the wild-type and variant sequence. Plasmids were transiently transfected into HEK-293 cells using Lipofectamine, and incubated for up to 48 h in Dulbecco’s Modified Eagle Medium (DMEM) supplemented with 10% fetal bovine serum at 37 °C and 5% CO_2_.

RNA was isolated using TRI Reagent^®^ and further purified using the RNeasy Mini Kit (Qiagen, UK, Catalogue No. 74106) which included a DNase digestion step. cDNA was synthesized from up to 4 μg of purified RNA using SuperScript™ reverse transcriptase (ThermoFisher Scientific, Catalogue No. 18091200) and subsequently amplified by Phusion high-fidelity polymerase (ThermoFisher Scientific, Catalogue No. F553) using primers designed to amplify all minigene transcripts. PCR products were visualized by electrophoresis on a 1–2% agarose gel and purified using the QIAquick Gel Extraction kit. Purified PCR products were Sanger sequenced and aligned to the reference sequence for the minigene vector using the SnapGene software suite and assessed for differences in splicing between wild-type and variant minigene constructs.

### Comparison with GTEx datasets

Variants identified in GTEx v7 datasets were cross-referenced with prioritized variants from our cohort. FASTQs were downloaded from the Database of Genotypes and Phenotypes (dbGaP) under the project accession phs000424.v8.p2 for GTEx control individuals carrying prioritized variants. RNA-seq datasets for samples carrying prioritized variants were identified, and the TPM value of the tissues available were considered. RNA-seq data from tissues with a TPM value > 5 were considered and FASTQ datasets were processed as described previously^49^. Read alignments were visualized in IGV and Normalized Read Count (NRC) and intron retention levels were quantified. NRC is calculated as the proportional usage of non-canonical splice junctions compared to canonical splice junctions for any given site. NRC and intron retention levels for individuals carrying prioritized variants were compared to ten control individuals in the GTEx dataset.

## Supplementary Information

Supplementary Information 1.

Supplementary Information 2.

## Data Availability

The datasets generated during and/or analyzed during the current study are not publicly available.
