# Supplementary material for: Comparison of in silico strategies to prioritize rare genomic variants impacting RNA splicing for the diagnosis of genomic disorders
Source: Sci Rep. 2021 Oct 18;11:20607. doi: 10.1038/s41598-021-99747-2 (PMC8523691; doi:10.1038/s41598-021-99747-2)
Supplement: Supplementary file 1 — Supplementary Information 1. [file 41598_2021_99747_MOESM1_ESM.pdf]

Box 1 - Case Example

The proband (Supplementary Fig. S1) was diagnosed in childhood with bronchiectasis; she is currently 54 years of age. Nasal nitric oxide levels were extremely low at 4 parts per billion (ppb, normal range ≤ 25 ppb) consistent with a diagnosis of primary ciliary dyskinesia (PCD)[1]. Three examinations of the proband's cilia with electron microscopy (EM) showed a significant proportion of static and dyskinetic cilia with a high ciliary beat frequency: 20.2Hz (95% CI = 19.8-20.5Hz); 22.0Hz (95% CI = 20.1-23.2Hz); and 20.1Hz (95% CI = 19.8–20.5Hz). EM histology showed normal dynein arms and microtubules with no ciliary disorientation, and conical ciliated protrusions were observed from epithelial cells. These findings are consistent with mutations in *DNAH11*, a recessive cause of PCD[2]. Previous genetic testing identified a heterozygous nonsense mutation in *DNAH11,* p.Tyr2870Ter.

We generated whole genome sequencing datasets through the 100,000 Genomes Project. Analysis of variants was restricted to the genomic region of *DNAH11* (chr7:21,582,833-21,941,457, GRCh37) and prioritized a candidate non-coding mutation, *DNAH11* c.6547-963G>A. The variant is absent from gnomAD, ClinVar, HGMD and other samples in the 100,000 Genomes Project; it was determined to be a 'variant of uncertain significance'.

We assessed *in silico* splicing scores for *DNAH11* c.6547-963G>A, and determined that it was 'disruptive' according to thresholds applied to SpliceAI and MaxEntScan scores (Supplementary Table S2). The variant had a *high* score from SpliceAI (0.67), but it was outside the regions of analysis for SPIDEX and S-CAP.

We performed targeted cDNA amplification from RNA extracted from whole blood (Supplementary Fig. S1). These analyses demonstrated aberrant splicing as a result of c.6547-963G>A, resulting in cryptic exon inclusion and the introduction of a premature stop codon, p.Ile2183Lysfs*15. The variant was determined to be 'likely pathogenic'. Segregation analysis demonstrated that p.Tyr2870Ter and p.Ile2183Lysfs*15 are present in a compound heterozygous state in the proband (Figure S1), and were reported as the cause of PCD.

Supplementary Tables and Figures

Supplementary Table S1. Variants of uncertain significance investigated through functional splicing assays. Scores from each of the *in silico* prioritization algorithms are included along with a consensus approach and a novel weighted metric.

[external Excel spreadsheet]

Supplementary Table S2. Pairwise comparisons of the AUC for each *in-silico* prioritization tool. Displayed are the p-values for each comparison, calculated using the pROC R package (bootstrap test, 2,000 iterations). Significant differences in the AUC after Bonferroni correction are indicated by bold text, showing that SpliceAI and a consensus approach perform better than all other approaches. All approaches perform better than CADD alone.

	SpliceAI	SPIDEX	MMSplice	MaxEntScan	KipoiSplice	TraP	S-CAP	CADD	Consensus
SpliceAI	x								
SPIDEX	**4.61E-09**	x							
MMSplice	**5.35E-06**	0.05125	x						
MaxEntScan	**9.23E-08**	0.08028	0.9835	x					
KipoiSplice	**5.37E-05**	0.009853	0.69	0.6723	x				
TraP	**9.79E-05**	**0.005707**	0.5183	0.4253	0.7236	x			
S-CAP	**2.01E-10**	0.5741	0.09294	0.1365	0.06732	0.03671	x		
CADD	**2.20E-16**	**8.08E-05**	**1.69E-12**	**6.73E-12**	**2.13E-11**	**1.01E-10**	**3.39E-13**	x	
Consensus	0.2358	**1.80E-09**	**2.74E-05**	**8.37E-08**	**5.43E-08**	**1.09E-05**	**4.47E-10**	**2.20E-16**	x

Supplementary Table S3. Optimal thresholds and values calculated through ROC-AUC for 250 variants of uncertain significance which had received functional analysis through blood-based RNA analysis or synthetic minigene assays. We determined 80/250 variants to impact splicing (true positives) and 170/250 variants to not impact splicing (true negatives). ROC curves were created using the pROC ggplot2 package in R, and 95% confidence intervals were calculated using 2000 stratified bootstrap replicates. Optimal thresholds were selected based on the maximum Youden's J statistic, as calculated using pROC.

Prioritization Approach	Optimal Threshold	Specificity	Sensitivity	AUC (95% CI)
SpliceAI	0.145	0.9	0.9113924	0.9536 (0.9238-0.9771)
SPIDEX	1.84715	0.8882353	0.5443038	0.7305 (0.6570-0.8018)
MMSplice	0.2892759	0.8235294	0.721519	0.8081 (0.7459-0.8659)
MaxEntScan	1.715	0.7411765	0.8607595	0.8103 (0.7576-0.8653)
KipoiSplice	0.509906	0.9176471	0.721519	0.8299 (0.7638-0.8942)
TraP	0.4675	0.8176471	0.7974684	0.8428 (0.7858-0.8938)
S-CAP	0.00245211	0.8117647	0.6329114	0.7471 (0.6823-0.8075)
CADD	25.2	0.4470588	0.7088608	0.4716 (0.4004-0.5486)
Consensus	3.5	0.8647059	0.8860759	0.9375 (0.9106-0.9651)
Novel weighted metric (SpliceAI & Consensus)	0.6275	0.9117647	0.9240506	0.9635 (0.9413-0.9818)

Supplementary Table S4. Thresholds utilized for each of the *in silico* splicing algorithms to identify variants which were expected to impact splicing.

Tool	Threshold
SpliceAI	0.2
MaxEntScan	Increase or decrease of 1
SPIDEX	Increase or decrease of 5
TraP	
Non-coding	0.289
Coding	0.416
MMSplice	Increase or decrease of 2
KipoiSplice	0.95
CADD	
Exonic	7.39
5extended	0.005
3intronic	0.006
5intronic	0.006
Anything else	0.006
S-CAP	
Exonic	0.009
5extended	0.005
3intronic	0.006
5intronic	0.006
5core	0.033
3core	0.034

Supplementary Table S5. Variability in accuracy of splice prediction tools. Using the standard error of the mean (SEM), 95% confidence intervals (CIs) were calculated across the 2,000 bootstraps used to calculate model accuracy, as depicted in Figure 2c. We observed that, for all tools investigated, variability in accuracy was low across the bootstraps.

Approach	Mean accuracy	SEM	95% CI
CADD	0.3877	0.001340	0.3864-0.3890
Consensus	0.8679	0.0009484	0.8670-0.8689
KipoiSplice	0.7475	0.001224	0.7462-0.7487
MaxEntScan	0.7247	0.001279	0.7234-0.7260
MMSplice	0.7560	0.001193	0.7548-0.7572
S-CAP	0.7512	0.001201	0.7500-0.7524
SPIDEX	0.6963	0.001262	0.6951-0.6976
SpliceAI	0.9082	0.0008068	0.9074-0.9090
TraP	0.7807	0.001172	0.7796-0.7820

Supplementary Table S6. Summary of rare unique variants prioritized by each *in silico* splicing prediction tool/strategy.

Tool	Number of Prioritized Variants
CADD	16,110
SPIDEX	684
SpliceAI	674
MaxEntScan	3,115
MMSplice	259
KipoiSplice	224
TraP	2,024
S-CAP	6033
Consensus (4/8)	632

Supplementary Table S7. Number of rare unique variants prioritized using different levels of consensus between *in silico* splicing tools.

Consensus (*n* of 8 tools)	Number of Prioritized Variants	Number of 5' / 3' core variants
1	8,660	1
2	6,373	1
3	1,439	1
4	279	4 (1%)
5	102	3 (3%)
6	76	21 (28%)
7	82	52 (63%)
8	93	83 (89%)

Supplementary Table S8. Summary of SpliceAI variant scores by their overlap with other *in silico* splicing tools also prioritizing the variant. LCI = lower 95% confidence interval; UCI = upper 95% confidence interval.

Consensus	Median	*n*	LCI	UCI
SpliceAI alone	0.36	2	0.060	0.661
1 other	0.267	48	0.230	0.304
2 others	0.290	101	0.251	0.330
3 others	0.424	103	0.375	0.474
4 others	0.541	63	0.477	0.604
5 others	0.783	50	0.719	0.846
6 others	0.961	73	0.914	1
7 others	0.974	88	0.945	1

Supplementary Table S9. Summary of SpliceAI variant scores by the impacted region of the genome, as defined in Jagadeesh *et al.*[3]; LCI = lower 95% confidence interval; UCI = upper 95% confidence interval.

**Region**	**Median**	***n***	**LCI**	**UCI**
3core	0.982	63	0.941	1
3intronic	0.432	78	0.369	0.495
5core	0.973	84	0.947	1
5extended	0.621	84	0.571	0.671
5intronic	0.487	22	0.391	0.584
exonic	0.303	197	0.271	0.336

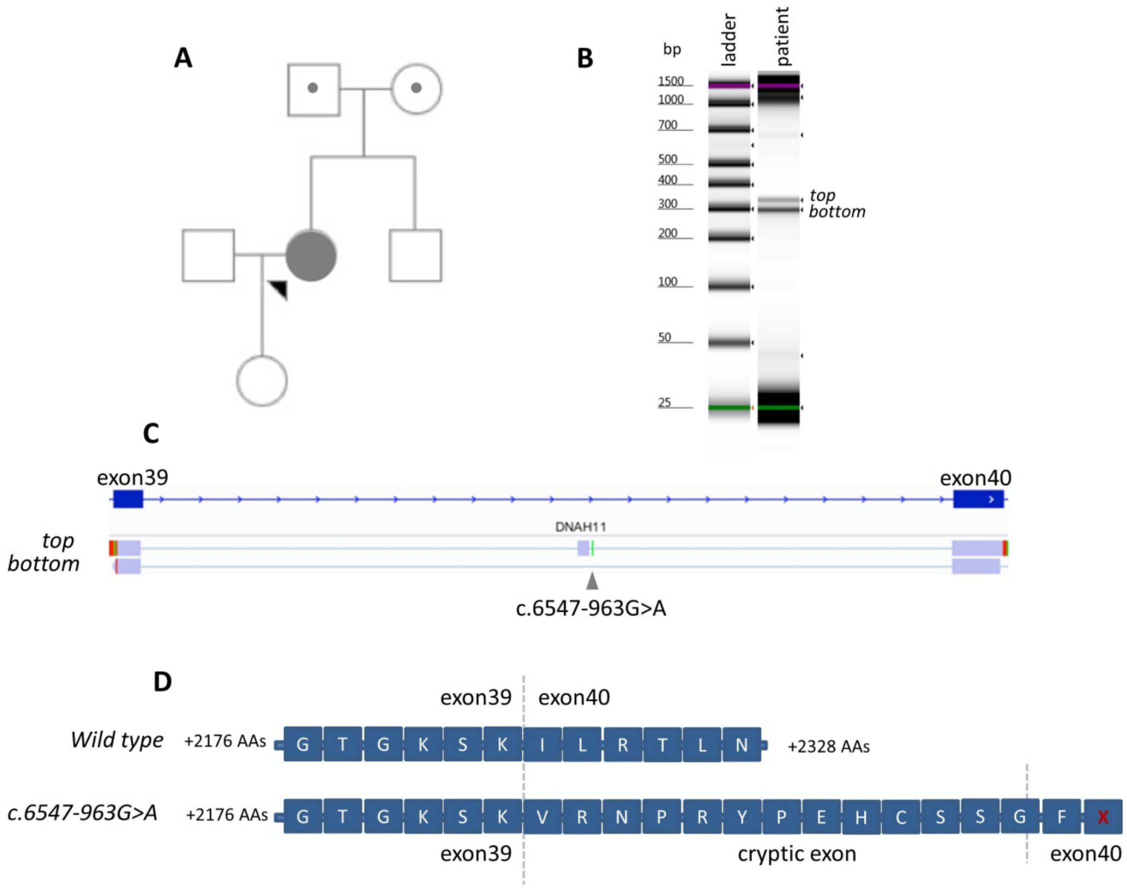

Supplementary Figure S1. *DNAH11* c.6547-963 G>A. (A) Family pedigree showing the proband and her unaffected father and mother who carry heterozygous alleles of *DNAH11* c.8610C>G and c.6547-963G>A, respectively. **(B)** Gel electrophoresis results for the proband, visualized using an Agilent 2200 Tapestation (original unaltered images are presented in supplementary figure S3). RNA was reverse transcribed after extraction from whole blood and then amplified using primers specific to exons 39 and 40 of the *DNAH11* gene (NM_001277115.1). The caption shows two distinct cDNA amplicons in the proband sample separated by ~40 base pairs. **(C)** Integrated Genomic Viewer snapshot of the alignment of sequencing products to the human reference genome (GRCh37) showing the introduction of a 38 base pair cryptic exon (chr7:21,746,318-21,746,355) as a result of c.6547-963 G>A. The *top* and *bottom* bands were sequenced after being cut from an agarose gel electrophoresis. **(D)** Impact of the cryptic exon on the translated protein. The cryptic exon shifts the reading frame and is expected to introduce a premature stop codon in exon 40, resulting in premature termination of protein synthesis, p.Ile2183Lysfs*15. Amino acids (AAs) are provided with single letter notations, with *X* indicating a stop codon. Vertical intersects indicate transition of the cDNA to the adjacent exon.

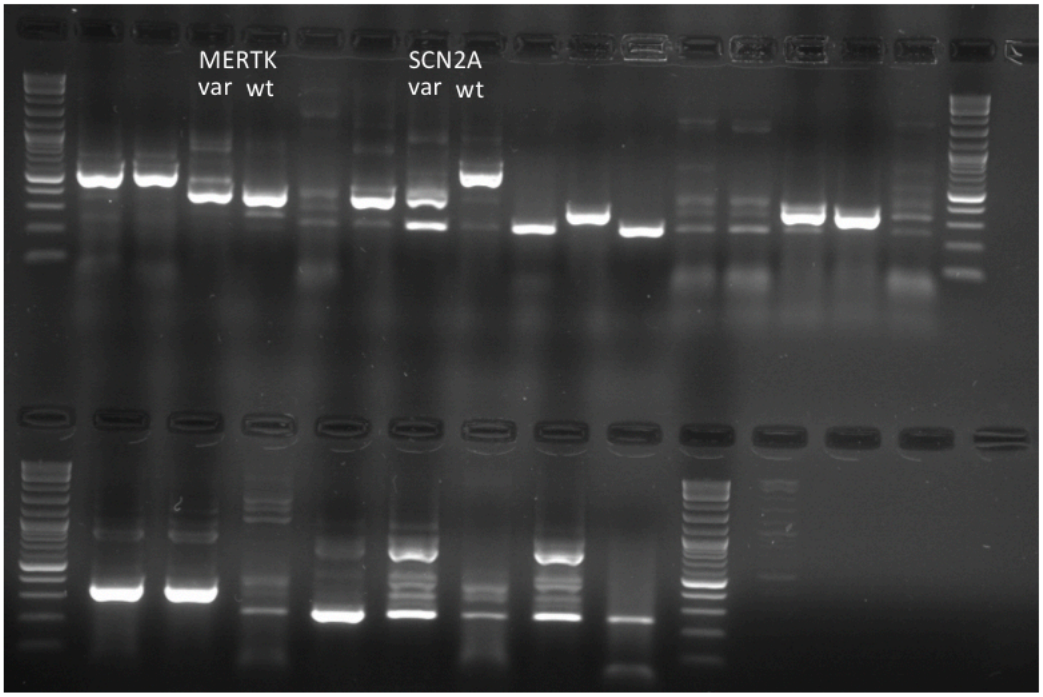

Supplementary Figure S2. Uncropped gel electrophoresis photographs for data used in Figure 1. The ladder used is the Invitrogen 1Kb plus ladder. The lanes used in figure 1 are indicated. Only the lanes indicated are relevant for MERTK c.2486+6T>A and SCN2A c.2919+3A>G.

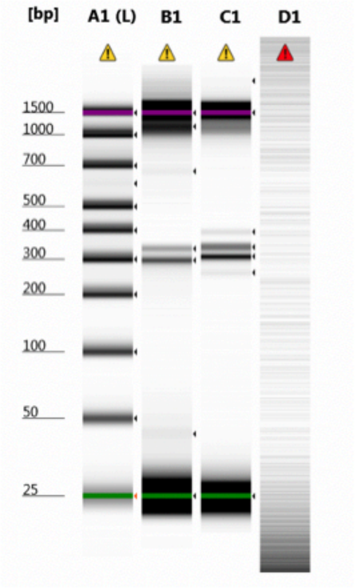

A1 – ladder
B1 - patient (used in Supp Figure 1)
C1 – patient (repeat)
D1 – empty

Supplementary Figure S3. Uncropped images from the Agilent Tapestation showing cDNA bands amplified from patient sample. The images shown are the default images from the Agilent 2200 Tapestation system, with band concentrations scaled to each individual sample.

Supplementary Bibliography

1 Walker, W. T., Jackson, C. L., Lackie, P. M., Hogg, C. & Lucas, J. S. Nitric oxide in primary ciliary dyskinesia. *The European respiratory journal* **40**, 1024-1032, doi:10.1183/09031936.00176111 (2012).

2 Schwabe, G. C. *et al.* Primary ciliary dyskinesia associated with normal axoneme ultrastructure is caused by DNAH11 mutations. *Human mutation* **29**, 289-298, doi:10.1002/humu.20656 (2008).

3 Jagadeesh, K. A. *et al.* S-CAP extends pathogenicity prediction to genetic variants that affect RNA splicing. *Nat Genet* **51**, 755-763, doi:10.1038/s41588-019-0348-4 (2019).
